# Provings and Clinical Observations with High Potencies

**Published:** 1893-08

**Authors:** Malcolm Macfarlan

**Affiliations:** Philadelphia, Pa.


### PROVINGS AND CLINICAL OBSERVATIONS WITH HIGH POTENCIES.

MALCOLM MACFARLAN, M. D., PHILADELPHIA, PA.

### THROAT.

### ARSENIC$^{6M}$.

*Throat very sore,* just as from an injury to her throat; swelling of the pharynx.

### APIS.

Sore throat; burning sensation with some swelling.

### ARUM-TRIF.$^{16M}$.

Sores on the throat; patches of ulceration, small and superficial.

Burning of pharynx and glottis; hoarseness.

### BARYTA-CARB.$^{CM}$.

Sore throat; dark-red, very large tonsils.

Cured chronic inflammation and enlargement of cervical glands; right side mostly affected; had resisted all other treatment; woman, æt. forty-five.

### BELL.$^{101M}$.

Feeling of lump in throat; dryness; swelling; nausea attends the feeling; throat constriction.

### BUFO.$^{45M}$.

Violent sore throat; pharynx so sore and swollen it is difficult to swallow food; verified frequently.

### BROMINE$^{CM}$.

Feeling of constriction in throat; an early symptom.

Caused very sore throat; later in proving.

### Bursa-past.$^{9M}$.

*Throat slightly sore;* dryness on swallowing; tonsillitis, tonsils swollen out on a line with angle of jaw.

Slight sore throat; upper part of his pharynx sore.

Stopped taking the medicine for eighteen days. On resuming it upper part of his throat became very sore as before.

Above remarks are the summary of many provings.

*Swelling of the throat* and face affects the left side mostly.

### Capsicum$^{1M}$.

Sore throat, hoarseness; dryness; burning pain on swallowing.

### Caulophyllum$^{5M}$.

*Hoarseness and loss of voice;* symptom not a prominent one.

### Cuprum-acet.$^{45M}$.

Throat very sore; pains her much; tries to raise phlegm, but raises little.

### Digitalis$^{CM}$.

Choking when she tries to swallow (spasm of glottis); only an occasional symptom.

### Erecthites$^{10M}$.

Throat slightly sore.

### Eupat-perf.$^{CM}$.

Throat dry; becomes at times hoarse.

### Gettysburg-salts$^{45M}$.

Hoarse, dry throat, as if scraped.

### Kali-bichr.$^{2C}$.

Cured speedily what is often called diphtheria; white patches on the tonsils; diphtheritic sore throat.

Cured congestion of the vessels; ulcers due to Mercury and syphilis.

### Kaolin$^{45M}$.

Sore throat in a slight degree.

### Lach.$^{6}$

Has produced very sore throat; sense of constriction with it.

### Kali-hyd.$^{CM}$.

Caused complete loss of voice; husky, croaking voice like croup.

### Mentha.$^{5C}$.

Very sore throat of a transient kind; mucous surface of the throat sensitive.

### Merc-viv.$^{101M}$.

Left side of throat sore; mostly prominent and reliable symptom; tonsils and pharynx very much swollen and inflamed.

### Iodine.

The inhalation of the vapors of Iodine, by placing a few drops of the tincture in a basin of boiling water, the child inhaling this under a sheet, has dislodged and expelled the membrane in two cases of apparent suffocation from diphtheria in trachea. Kali-bichr., 2C, given internally.

### Nitric-acid$^{5C}$.

Rapidly cured a case of syphilitic sore throat, ulcerative.

Sore throat with coryza and hoarseness; ulcers with well defined edges as if punched out.

### Pix-liquida.$^{5C}$.

Throat seemed quite sore, lasting several days—not a marked symptom.

### Petrol.$^{CM}$.

Throat has a burning sensation mostly on swallowing.

PALLADIUM$^{20M}$.

Slight sore throat.

PHOSPHORUS$^{CM}$.

Dryness in the fauces; burning sensation low down in pharynx; spitting and hawking.

Choking, constant desire to swallow, with burning sensation at epigastrium.

PLANTAGO-MIN.$^{11C}$.

Choking sensation; constriction of the throat.

RHUS-TOX.$^{105M}$.

Sore throat, tonsillitis; considerable swelling.

SULPH-AC.$^{5C}$.

Dryness in throat; soreness, nausea attending it.

TARAXACUM$^{67M}$.

Constriction felt on swallowing; feeling as if the throat was too small; a very marked symptom; muscular soreness in neck, shoulders, and chest.

THERIDON$^{5C}$.

Sore throat for a week; difficulty in swallowing.

CROTON-TIG.$^{2C}$

Causes sore throat; felt mostly near the larynx, and while swallowing.

FORMICA$^{45M}$.

Felt as if an abscess was forming in her tonsils; left side worse than right; throat was very sore on swallowing or drinking.

### VERBASCUM$^{45M}$.

Cured quickly a very severe pain in pharynx on swallowing; chronic case; accompanied by dyspeptic symptoms.

### APPETITE.

### ACTÆA-RAC.$^{5}$.

Complete loss of appetite.

### ARSENIC$^{6M}$.

No appetite; nausea.

### COCHLEARIA$^{10M}$.

Very hungry, unnatural craving for food.

### CUPRUM-ACET.$^{95M}$.

*Taste as of something putrid;* nausea; no appetite.

### FILIX-MAS.$^{1M}$.

He spits up or raises some food soon after taking it; weight at epigastrium; giddiness; sick stomach.

### IODINE$^{17M}$.

Nausea; foul breath.

### KALI-HYD.$^{CM}$.

Eructations; loss of appetite.

### MERC-VIV.$^{101M}$.

Complete loss of appetite; coated tongue.

### NUX-VOM.$^{94M}$.

Vomits frequently; had to force himself to eat.

### POLYGONUM$^{42M}$.

Burning sensation at epigastrium with nausea.

### PSORIN$^{42M}$.

Appetite much better; she has to get up in the middle of the night to eat; desire for crackers or bread.

### PULSATILLA$^{CM}$.

Giddy, weak, sick at stomach, no appetite.

### SAMBUCUS$^{45M}$.

Complete loss of appetite.

Everything nauseates; giddiness a marked symptom.

### SULPHUR$^{CM}$.

The peculiar faint and hungry feeling which comes over one, particularly at night, when proving Sulphur high, is very remarkable and characteristic; they want food, usually bread, at once, or they think they will suffer or perish; it is a sensation of faintness with hunger and longing for food.

Great gnawing hunger; thinks she will die unless she gets something to eat quickly.

### SEPIA$^{45}$.

Cured permanently an empty hungry feeling in epigastrium; severe dyspeptic symptom; had it for years.

### STOMACH.

### ARSENIC$^{6M}$.

Not much thirst; eructation after eating.

### APOCYNUM$^{6M}$.

Feels hungry, and when she tries to eat pain comes in epigastrium; food apparently sours.

Vomits very often; sick at stomach; two last symptoms appear after several days' proving.

### AGARIC.$^{2M}$.

Nausea; vomits often, and a great quantity of fluid.

### ACTÆA-RAC.$^{5C}$.

Nausea; uncertain feeling in epigastrium.

### OPIUM$^{69M}$.

Nausea; foul breath; coated tongue.

### ARANEA$^{45M}$.

Vomits frequently; every day of the proving.

### ARUM$^{16M}$.

Feels sick or qualmish; does not vomit; slight burning sensation at epigastrium.

### BELL.$^{101M}$.

Vomiting began on second and third day.

Sick and fainty very often; very pale; cannot keep much on her stomach.

Cannot keep food in any quantity on his stomach; passes water freely; feels weak; unable to work or think; head is light; nausea a prominent and reliable symptom of potentized Bell. It has cured frequently the persistent vomiting of pregnancy when nothing else would.

Great thirst; does not care much for food; wants to drink, it does not satisfy; burning sensation in pit of stomach; taking fluid increases distress in epigastrium; in the provers of Bell. high, most of them complain of a pressive pain running back of the middle of the sternum.

### BRY.$^{103M}$.

Pain in the epigastrium with slight nausea.

### BURSA-PAST.$^{9M}$.

Pain in his stomach and feels generally as if he would be sick; feels like vomiting.

Crampish pain in his stomach as a later symptom; coming and going, only lasting a short time.

CHELIDONIUM$^{5C}$.

Vomited his meals several times; chilly sensations afterward.

COLCHLEARIA$^{10M}$.

Occasional cramps about the umbilicus.

CUPRUM-ACET.$^{45M}$.

*Cramps in the stomach* and small bowels; severe cutting pains extending to inguinal region; nausea; coldness; thirst; vomits often.

DRACONTIUM$^{10M}$.

Constant eructation of wind and rumbling of wind in abdomen.

EUPION$^{2C}$.

Stomach felt sore and swollen; abdomen not sore on pressure.

FERRUM-ACET.$^{2C}$.

Deathly sick at stomach with vomiting. This symptom appeared three days after taking the medicine; never happened so before.

GELSEM.$^{CM}$.

Vomiting occasionally; stupid feeling about head.

HYPERICUM$^{45M}$.

Great pain in stomach; nausea, aggravated by eating.

HAMAMELIS$^{10M}$.

Sensation of trembling in stomach; not a permanent symptom.

HYPERICUM$^{45M}$.

Constant eructations; great fullness about abdomen; highly curative for this symptom.

### Kreosote$^{CM}$.

Constant nausea; disposition to vomit. The most reliable general remedy in cholera infantum, and many forms of persistent vomiting; relieves pain in ulcer and cancer of stomach.

### Ledum$^{45M}$.

Slight nausea.

### Lilium-tig.$^{45M}$.

Feels like vomiting when she presses her epigastrium. Otherwise slight nausea.

### Linaria 3d decimal.

Taken in water every hour, for three days, after, or on the fourth day, caused giddiness, sick stomach, vomited three times; bowels loose; rumbling noise in abdomen; no marked urinary or other symptoms as I had expected.

### Mississquoi$^{45M}$.

Nausea; feels sick and weak.

### Muriatic-acid$^{5C}$.

Slightly nauseated, mucous surfaces of mouth and throat sensitive.

### Nux-vomica$^{94M}$.

Relieved severe pain in epigastrium; felt as if the mucous membrane of the stomach were sore.

### Nitric-acid$^{5C}$.

Raises a small amount of food, after eating mostly.

### Palladium$^{20M}$.

Slight nausea.

### Petrol$^{CM}$.

Constant thirst, with distress in region of stomach, and disposed to diarrhœa and cough.

### PHOSPHORUS$^{CM}$.

Burning pain and cramps in stomach. The burning in the stomach extends up the œsophagus, with feeling of constrictive dryness in fauces; bloated stomach and abdomen. These are very constant and reliable symptoms, always noticed in provings.

### PLANTAGO-MINOR$^{11C}$.

Pain in the lower part of his abdomen.

### PSORINUM$^{42M}$.

Occasional pain across the epigastrium more constant in the spleen.

### PSORIN$^{42M}$.

Troubled greatly with wind, interferes with breathing.

### POLYGONUM$^{42M}$.

Very sick at stomach. A variable symptom—apt to come and go.

### SARACENIA$^{2M}$.

Hungry. Very nervous with it.

### STANNUM$^{38M}$.

Great weakness at the epigastrium and then gets or feels hungry, but cannot eat; feels as if she would faint; feels entirely gone at epigastrium.

### SULPHUR$^{CM}$.

Caused vomiting. Only an occasional symptom.

### SYMPHYTUM$^{15C}$.

Pains across his epigastrium from one side to the other, worse opposite the spleen on walking; when sitting worse about the navel, griping pain about the umblicus often occurs.

### TRIFOLIUM-PRETENS.

Sensation of gnawing, rumbling, and movement in abdomen; fermentation, eructation. Pain in bowels. Saliva profuse and bitter.

### UVA-URSA.

Slight nausea.

### WOORARI$^{90M}$.

Frequent nausea; vomiting of bile; no appetite; foul breath; soreness in stomach.

### ABDOMEN.

### AGARIC.$^{2M}$.

Soreness to touch in abdomen below navel.
A good deal of twisting pain about the umbilicus.

### ADEPS$^{1M}$.

Cramps in stomach; cramps about navel.

### APUINO$^{69M}$.

Very bad pains in the bowels.

### ARSENIC.

Highly curative in abdominal dropsy; it relieves when a cure is impossible.

### BURSA-PAST.$^{9M}$.

Pain between the end of sternum and umbilicus like needles or like a shock from battery.

### COLLINSONIA$^{45M}$.

Weight in epigastrium; inveterate case of dyspepsia complicated with hemorrhoids, cured.

